# Durability and Service Life Prediction of Fluorocarbon Elastomer under Thermal Environments

**DOI:** 10.3390/polym14102047

**Published:** 2022-05-17

**Authors:** Pyoung-Chan Lee, Su Young Kim, Youn Ki Ko, Jin Uk Ha, Sun Kyoung Jeoung, Ju-Yub Lee, Minsu Kim

**Affiliations:** 1 Materials Technology R&D Division, Korea Automotive Technology Institute, Cheonan-si 31214, Chungnam, Korea; sykim1@katech.re.kr (S.Y.K.); ykko@katech.re.kr (Y.K.K.); juha@katech.re.kr (J.U.H.); skjeoung@katech.re.kr (S.K.J.); 2 Reliability R&D Division, Korea Automotive Technology Institute, Cheonan-si 31214, Chungnam, Korea; leejy@katech.re.kr; 3 FL Material Research Team, Hwaseung Material Co. Ltd., Yangsan-si 50592, Gyeongnam, Korea; mskim01@hsrna.com

**Keywords:** service life, fluorocarbon elastomer, accelerated life test, durability, automotive

## Abstract

This study investigated the service life prediction of fluorocarbon elastomers that are used in automotive vapor fuel hoses under thermal environments. The changes in mechanical properties such as the tensile strength, elongation, compression set (CS), and hardness according to thermal aging were investigated for two types of ternary fluoroelastomers. Destructive tests of the tensile strength and elongation showed large variations in the mechanical properties under the same condition because there is no continuity of samples. In contrast, nondestructive tests of the CS and hardness showed little variations in the mechanical properties under the same condition. The elongation, CS, and hardness were selected as the physical parameters for service life prediction as they showed a tendency according to the aging temperature, which is an accelerating factor. The effective activation energy derived using each physical parameter was 74.91–159.6 kJ mol^−1^, and the service life was 17.8–140 × 10^3^ h based on B_10_. In this study, hardness, which has a small deviation between samples, is considered appropriate as mechanical parameter for predicting the service lifetime.

## 1. Introduction

Rubber materials are widely used in the automotive, railroad, and construction industries. Fluoroelastomers are rubbers in which fluorine has been introduced; they show improved properties at high temperatures [1,2,3]. They also have excellent resistance to organic oils owing to the polarity of the molecular composition, making them applicable in many fields. Moreover, the curing system of fluoroelastomers results in excellent thermal stability, flexibility, and resilience at high temperatures [1,2,3]. Owing to these characteristics, fluoroelastomers are widely used in power transmission parts and fuel systems for motor vehicles [1,2,3]. Fluoroelastomers are of two main types: fluorocarbon elastomer and inorganic fluoroelastomer. Fluorocarbon elastomers (hereafter called simply fluoroelastomers) are the most common fluoroelastomers; they consist of monomers with carbon-carbon bonds having fluorinated functional groups with varying amounts of fluorine [1,3].

Polymers including rubber are exposed to various environmental factors such as temperature, humidity, moisture, mechanical stress, or combinations of them [3,4,5,6,7]. These factors negatively impact the properties or performance of polymers and affect the service life or durability of parts in which they are used. Therefore, polymers should ideally exhibit excellent physical, chemical, and mechanical properties even after several years of use. However, their initial performance often changes with operation over time. The properties of elastomers also undergo various changes in service environments, and the elastomers may become impossible to use owing to excessive hardening, cracking, or other surface damage [3,5,8]. The durability of polymers is crucial to manufacturers and users of plastic products, and therefore, the degradation process of polymers is attracting research attention [5]. The service life of polymers at room or elevated temperatures is the key factor determining the allowable life span (warranted by the manufacturer). After this time, the material reaches the threshold of measured value at service temperature [5,9]. The storage life varies depending on the product storage conditions, elastomer type, product type (e.g., seal, gasket, tube, tires), and application (e.g., automotive, aerospace, ship) [5].

Many automotive parts are designed and produced for a service life of several years or decades. In the design of such parts, methods for mathematically describing and predicting the degradation mechanism of materials are needed. If a material’s degradation behavior in a given service environment is insufficiently understood, the potential of the material cannot be fully exploited, and the general product cost may increase because of oversizing components. Moreover, recall and repair costs may increase sharply owing to an increase in the product defect rate. The service life prediction of polymers offers the advantage of identifying and isolating the material properties in response to critical damage. Therefore, accurately predicting the service life of materials used in automotive parts is crucial in safety terms [4,5].

An accelerated aging test is conducted by intensifying the factors that accelerate aging by using laboratory equipment to create conditions that exceed the normal service range [3,4,5,10]. The service life is predicted by modeling the changes in important mechanical properties, including the shore hardness, tension, impact, modulus, thermal decomposition, compression set (CS), and fatigue, under accelerated aging and setting reliability criteria [3,4,5,6,11,12,13,14,15].

This study investigated the service life prediction of fluoroelastomers for an automotive vapor fuel hose based on changes in various physical parameters under thermal environments. Previous studies [3,16] used shore A under thermal environments as a physical parameter to derive an accelerated service life model based on the Arrhenius relation by applying the time–temperature superposition principle. In this study, the service life was predicted, and the activation energy was estimated based on changes in various physical parameters under thermal environments.

## 2. Materials and Methods

### 2.1. Materials

G-558 grade (tube 1; Daikin Co., Osaka, Japan) and DTR-5930GE grade (tube 2; Dowhon, Chengdu, China) ternary fluoroelastomers that are copolymers of vinylidene fluoride (VF2), hexafluoropropylene (VF6), and tetrafluoroethylene (VF4) were used in the experiments [16]. The samples of fluoroelastomers were fabricated and supplied by Hwaseung Materials Co. (Yangsan-si, Korea).

### 2.2. Aging Test

The thermal aging test was conducted using a forced-air-type oven (ThermoStable OF-50, Daihan Scientific Co., Wonju-si, Korea) at temperatures of 160, 175, 190, and 200 °C. The range of aging temperature was determined by considering the vapor tube hose operating temperature and the heat resistance (200 °C or lower) of the materials (e.g., fluoroelastomer, additives).

### 2.3. Analysis

Thermogravimetric analysis Fourier transform infrared (TGA-FTIR) analysis was conducted using PerkinElmer’s TGA 4000 and Spectrum Two (Waltham, MA, USA). The TGA measurement conditions were as follows: 30–900 °C section with 20 mL min^−1^ of N_2_ gas at a heating rate of 10 °C min^−1^. The surface characteristics before and after aging were measured using optical microscopy (VHX-2000, Keyence, Osaka, Japan) and atomic force microscopy (AFM, NX10, Park Systems, Suwon-si, Korea). Mechanical properties including the tensile strength and elongation were measured using a universal testing machine (UT-100F, MTDI Korea, Daejeon, Korea). The hardness (shore A, ASTM D2240) was measured using a digital durometer (DD4-A, Kobunshi Keiki, Kyoto, Japan), and the CS was measured in accordance with ASTM D395.

## 3. Results and Discussion

### 3.1. TGA-FTIR Analysis

**Figure 1:**
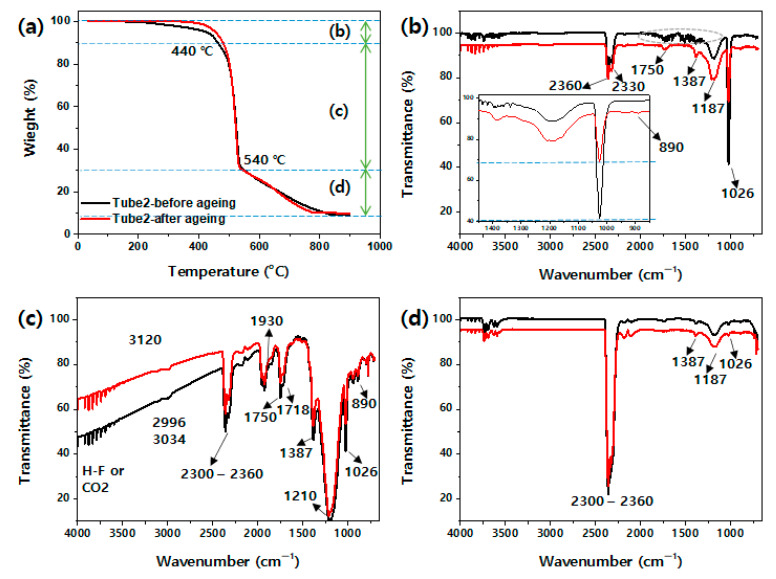
TGA-FTIR spectra of fluoroelastomer (tube 2) thermally degraded at 200 °C (1000 h): (**a**) TGA graph, (**b**) FTIR spectra at 440 °C, (**c**) FTIR spectra at 510 °C, and (**d**) FTIR spectra at 710 °C.

A previous study [3] conducted an FT-IR analysis of the degradation of a fluoroelastomer used in a gasket. The results revealed a change in the peak by the thermo-oxidative scission reaction of dehydrofluorinization. The present study conducted TGA-FTIR analysis to examine the structural changes caused by accelerated aging in fluoroelastomers. Figure 1a shows the TGA results before and after the aging of tube 2. Figure 1 shows that in the sample before aging, pyrolysis started after 200 °C, and in the sample after aging, pyrolysis started after 350 °C, and it progressed rapidly until 540 °C. The FT-IR measurements of the gas generated by pyrolysis were performed for the initial pyrolysis section until 440 °C (Figure 1b), the section until 540 °C in which the pyrolysis rate changes (Figure 1c), and the remaining section (Figure 1d). As can be seen in Figure 1b, noise caused by moisture was observed in the sample before aging (green dotted oval). The peak at 2300–2360 cm^−1^ was generated by CO_2_ gas [17]. The peaks at 1387 and 890 cm^−1^ were generated by CF and CF_3_, respectively, and those at 1187 and 1026 cm^−1^ were generated by CF_2_ [3,18,19]. The peak at 1750 cm^−1^ was attributable to C=C and COO^–^ generated by the oxidation response [19]. The peaks at 2920 and 2852 cm^−1^ corresponding to the C–H structure of the fluoroelastomer did not appear, possibly owing to pyrolysis. The peak at 1026 cm^−1^ significantly decreased owing to thermal aging. A comparison with the TGA result in Figure 1a indicates that the unreacted fluorine-based monomers or oligomers with low molecular weight underwent additional reactions under high-temperature conditions. The pyrolysis before aging was considered to have started after 200 °C owing to the unreacted material. Moreover, the pyrolysis temperature appeared to have increased owing to the increase in molecular weight due to the additional hardening of the unreacted material after aging.

Figure 1c shows the FT-IR measurement result of the gas generated in the section where rapid pyrolysis occurred. Various structural peaks corresponding to the fluoroelastomer and peaks of CO_2_ gas are seen in Figure 1c, indicating that the main component of the fluoroelastomer was pyrolyzed. The peaks at 1718 and 1930 cm^−1^ were attributed to the vibration of C=O [19,20]. Figure 1d shows the FT-IR measurements of the gas generated after 540 °C; CO_2_ gas was seen to be the main component.

### 3.2. Surface Morphology

**Figure 2:**
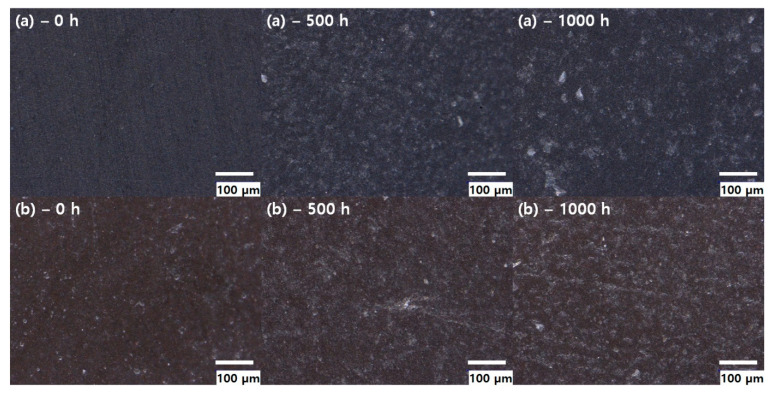
Optical microscopy image of fluoroelastomer with different thermal aging times: (**a**) tube 1 and (**b**) tube 2.

The deterioration of the fluoroelastomer was visually observed using an optical microscope. Figure 2 shows optical microscopy images of the fluoroelastomer with different aging times. As can be seen in Figure 2, the surface condition of the fluoroelastomer appeared smooth at first (as shown in Figure 2(a)–0 h and 2(b)–0 h) and then became rough with time. In general, all materials showed surface deterioration, including micro-cracks. The appearance of micro-cracks of fluoroelastomer appears to be caused by the migration of substances with a low molecular weight. These changes in surface properties may affect the crack initiation and ultimate failure [21,22].

**Figure 3:**
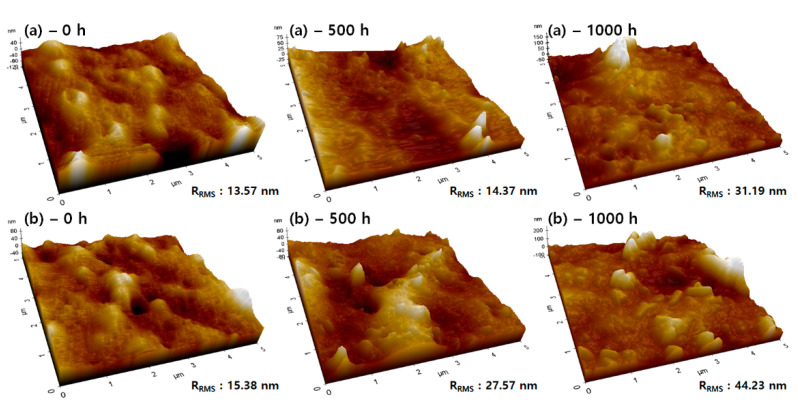
AFM image of fluoroelastomer with different thermal aging times: (**a**) tube 1 and (**b**) tube 2.

Figure 3 shows the AFM data of the fluoroelastomer (tube 1) before and after aging. As can be seen in Figure 3, the aged sample has a rough surface with higher peaks and deeper valleys compared to the sample before aging. The average surface roughness value (S_a_, 10.8 nm) and root mean square roughness (S_RMS_, 13.57 nm) before aging increased owing to the increased surface roughness after aging (S_a_ 15.3 nm, S_RMS_ 31.19 nm). This result indicated that the fluoroelastomer is not resistant to high temperature and surface degradation after the aging test.

### 3.3. Mechanical Properties

**Figure 4:**
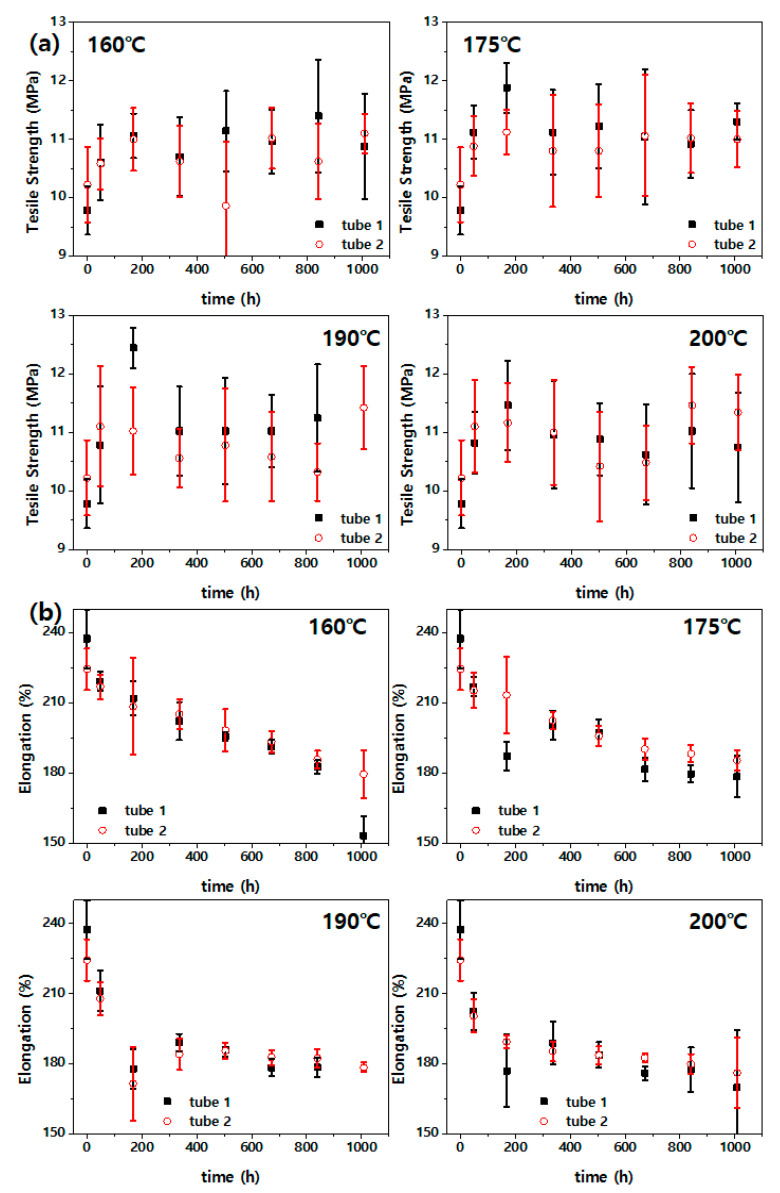
(**a**) Tensile strength and (**b**) elongation of fluoroelastomer against aging time at different aging temperatures.

Figure 4 shows the change in the tension properties (i.e., strength and elongation) of fluoroelastomer samples aged under various high temperatures. Figure 4 shows that the mechanical properties of the fluoroelastomer gradually changed with the aging time. As can be seen in Figure 4a, the tensile strength shows very large deviations, and it is difficult to clearly distinguish a trend according to the aging time. Tube 1 showed average tensile strengths of 11.40 and 10.88 MPa after aging for 840 and 1008 h at 160 °C, respectively, and 11.02 and 10.74 MPa after aging for 840 h and 1008 h at 200 °C, respectively. Thus, it is difficult to specify the aging temperature as an acceleration variable because it results in insignificant differences in the tensile strength. This is considered attributable to the large variations of properties between samples resulting from the lack of continuity in the properties of the same samples because the tensile test destroys the sample. As a result, this seemingly makes it difficult to reliably infer the time required to reach the critical point of the tensile strength. As shown in Figure 4b, the elongation initially decreased sharply and then decreased slowly. This can be considered to show a tendency according to the aging time with aging temperature. However, as with the tensile strength, the variations of properties among the samples are large.

**Figure 5:**
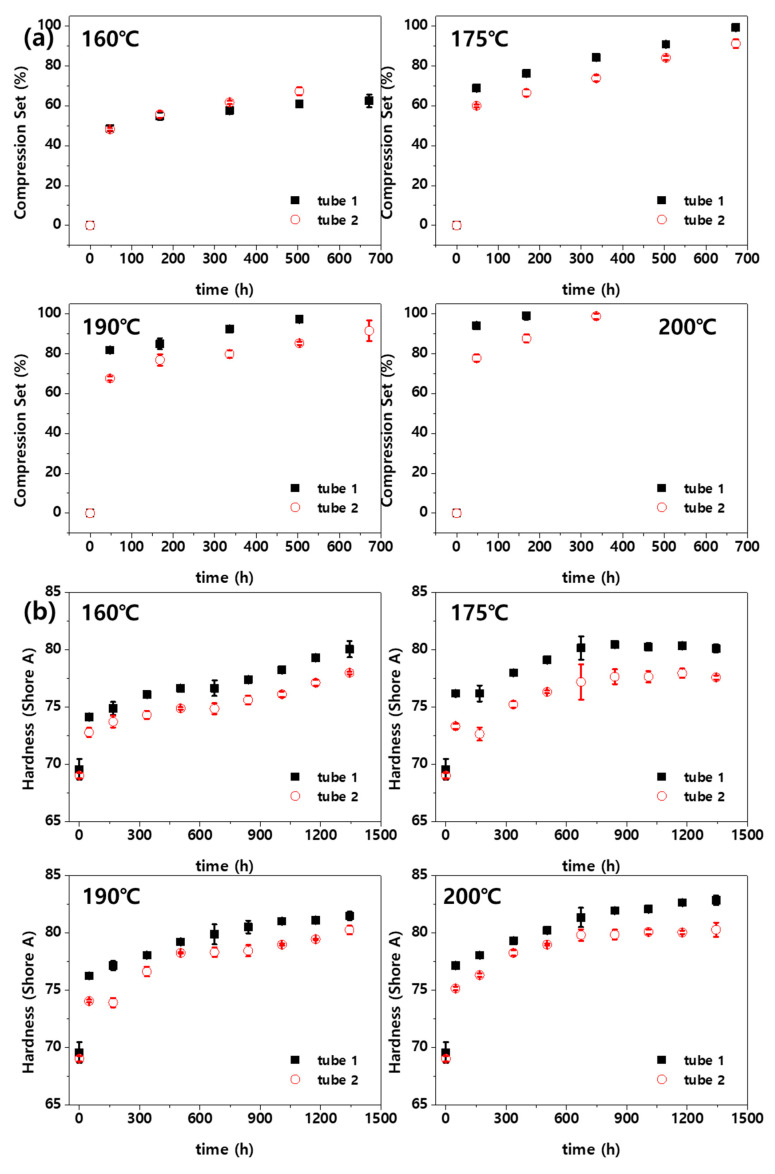
(**a**) CS and (**b**) hardness of fluoroelastomer with aging time at different aging temperatures.

Figure 5 shows changes in the CS and hardness of the fluoroelastomer at various high temperatures. Figure 5a shows changes in the CS according to the aging time. CS indicates the elasticity (or resilience) of rubber materials. Most fluoroelastomer-hardening systems have cross-links and are stable against heat. Therefore, major network breakdown is rare in a short-term degradation test [1]. However, if the material is exposed to a high temperature for a long time, CS increases as the material loses resilience due to thermo-oxidative reaction. The CS changed rapidly and then slowly increased. The results for aging temperatures of 190 °C and 200 °C showed that the CS value reached 100% in the middle of the experiment. Because the resilience is no longer effective in this case, the subsequent experimental results were considered meaningless and were not plotted. Unlike the properties shown in Figure 4, those shown in Figure 5 did not exhibit significant deviations among the samples. This appears to be because the CS and hardness tests are nondestructive, and the values of the properties are continuous over the aging time. Figure 5a shows that the CS value of tube 1 at 160 °C is smaller than that of tube 2. However, at higher temperatures, the CS value of tube 1 is larger than that of tube 2. This implies that tube 1 has higher resilience at low temperature, whereas tube 2 has higher resilience at high temperature. Moreover, the resilience of tube 1 can be inferred to decrease sharply at temperatures higher than a certain value. This also affects the service life of a material. Figure 5b shows that, as in previous studies [3,16], the hardness rapidly increased in the early aging phase and then slowly increased. Furthermore, the hardness increase changed rapidly as the aging temperature increased. The time required for tube 1 to reach a hardness change rate of 10% was 577–805 h at 160 °C, 158–218 h at 175 °C, and 87–97 h at 200 °C.

### 3.4. Service Life Prediction

Because the material properties changed with the aging temperature and time, as shown in Figure 5, the Arrhenius model, which is a function of temperature, was selected as the acceleration model. The service life model was examined using the Minitab program. As shown in Figure 4a, the changes in tensile strength with temperature did not show any significant trend. Therefore, the tensile strength seems unsuitable as a physical parameter for service life prediction. A review of the exponential distribution, Weibull distribution, and log distribution revealed that the Weibull distribution was the most suitable service life model. The measure of reliability is the reliability function as a function of service time *t*. The cumulative distribution function of the Weibull distribution, which is most widely used for reliability, can be expressed as [6,16]

(1) $$F\left(t\right)=1-\exp\left[-{\left(t/\eta \right)}^{n}\right],$$

where *F(t)*, *n*, and *η* denote the cumulative distribution function, shape parameter, and scale parameter, respectively.

The time-dependent speed (*k*) in the accelerated aging test of plastics can be represented by the Arrhenius relation [6]. Furthermore, the scale parameter *η* of the service life distribution model can be replaced by the service life property in the accelerated model. Consequently, the scale parameter of the Weibull distribution can be expressed as being equal to the time-dependent speed (*k*) [6,16]:

(2) $$k=k_{0}\exp\left(\frac{-E_{a}}{RT}\right)=\eta \;,$$

where *k_0_* denotes a constant; *E_a_* denotes the activation energy (J mol^−1^); *R* denotes the gas constant (8.314 J·K^−1^·mol^−1^); and *T* denotes the absolute temperature (K). As a result, the cumulative distribution function in Equation (1) can be expressed as follows [6,16]:

(3) $$F\left(t\right)=1-\exp\left[-{\left(\frac{t}{k_{0}\exp\left(\frac{-E_{a}}{RT}\right)}\right)}^{m}\right],$$

**Figure 6:**
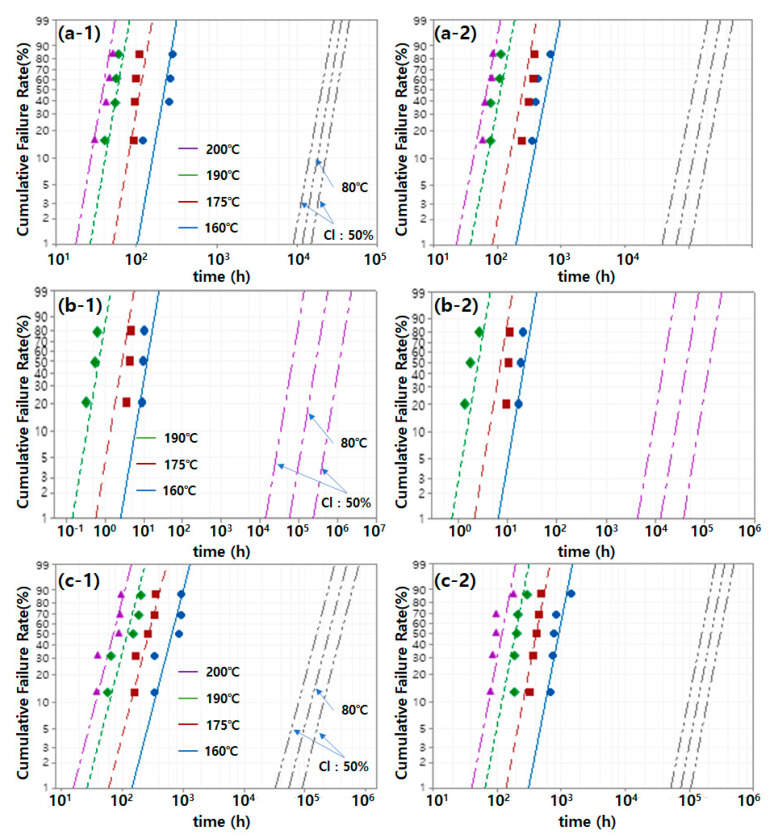
Arrhenius–Weibull probability plot of the (**a**) elongation, (**b**) CS, and (**c**) hardness of the fluoroelastomer under accelerated aging.

Figure 6 shows a straight line that has been fitted according to the acceleration parameter based on the failure time of the material derived from the results in Figure 4 and Figure 5. The three lines on the right side in each graph represent the estimated straight line assuming an operating temperature of 80 °C and 50% confidence interval (CI). Various mechanical properties were analyzed to obtain mechanical property data for accelerated life modeling. The “equivalent aging time,” which associates the result of accelerated thermal aging with the temperature profile of the actual use environment, can be calculated. Furthermore, the acceleration/prolongation factor, which associates the aging times at different aging temperatures, can be calculated [12]. As described in Figure 4a, the tensile strength was considered unsuitable as a parameter for service life prediction because it was difficult to infer its trend according to the aging temperature and aging time. As discussed with respect to Figure 4b, the estimated straight line in Figure 6a and the actual result (points in the graph) showed a difference owing to the large variation of the elongation. In Figure 6b, the CS at 200 °C changed within a short time (as shown in Figure 5a), resulting in insufficient data; thus, it was not used for service life prediction. As shown in Figure 6b, the threshold point arrival time at high temperature was very short. In this case, the experimental error could have a large effect.

The B_x_ life represents the time when the probability of failure is x%. In this study, the B_x_ life of the fluoroelastomer can be obtained from the cumulative distribution function in Equation (3) and Figure 6, and it was calculated using the Minitab software. Table 1 summarizes the B_x_ life of the fluoroelastomer derived from the accelerated aging results by physical parameter. As shown in Figure 6b, tube 1 showed higher resilience than tube 2 at low temperature (below 160 °C). As a result, it showed a longer service life in the 80 °C atmosphere. As shown in Table 1, the B_x_ life derived from the CS and hardness was similar for tube 1. However, the B_x_ life derived from the Cs was much shorter than that derived from the hardness for tube 2. The resilience of rubber is affected by various factors such as the cross-linking degree of the material and molecular structure. The effect of the aging mechanism of tube 2 by heat on the CS requires further research. Unlike the CS result, as shown in Figure 6c, the service life of tube 2, which showed excellent hardness in all experiments, was longer than that of tube 1.

Table 2 summarizes the activation energy inferred from the CDF in Equation (3) and Figure 6. Notably, every activation energy is an “effective value” estimated under the assumption that it is the dominant aging mechanism that can be approximated by thermal activation of the Arrhenius type and can be identified for each property. Table 2 shows that the effective activation energy derived from CS is higher than that derived from hardness. This seemed attributable to the difference in the aging mechanism of each material property [21]. The used environment and major properties required differed depending on the application and applied part. The selection of a physical parameter in consideration of the service environment of the applied part was crucial because the service life of each material differed with the physical parameter. The results obtained in this study suggested that the hardness was more suitable than the CS, which has a large effect on the error because a short time is required for reaching the critical point at high temperature. Furthermore, although the tensile strength and hardness showed similar effective activation energies, the hardness had a smaller error than did the elongation (which showed large errors among samples) and was therefore considered a more suitable parameter.

## 4. Conclusions

This study aimed to select the most suitable method for evaluating the service life and reliability of fluoroelastomer parts used in the automotive vapor fuel hose as well as for shortening the verification time required for selecting the appropriate material. The activation energies of different physical properties were estimated by assuming the existence of Arrhenius-type temperature behaviors from the aging time dependency of different material properties at different aging temperatures. Assuming the Arrhenius relation, the acceleration coefficient or service life for a random temperature profile can be calculated by using the concept of “equivalent aging time.” The results showed that hardness was a more suitable physical parameter for predicting the service life. These findings should be helpful in securing the long-term durability and reliability of fluoroelastomer parts.

## Figures and Tables

**Table 1:** Summary of B_x_ life of fluorocarbon elastomer for various physical parameters as calculated from cumulative density function using accelerated thermal aging test.

Physical Parameter	B_x_	Service Time (×10^3^·h)	
		Tube 1	Tube 2
Elongation	B_0.1_	7.60	35.0
	B_1_	11.6	63.7
	B_10_	17.8	117
CS	B_0.1_	24.8	6.47
	B_1_	58.4	12.7
	B_10_	140	25.1
Hardness	B_0.1_	23.7	40.7
	B_1_	53.9	73.5
	B_10_	124	134

**Table 2:** Effective activation energies for different physical parameters.

Method	Physical Parameter	E_a_ (kJ mol^−1^)	
		Tube 1	Tube 2
Thermal	Elongation	74.91 ± 4.331	91.69 ± 8.942
	CS	159.6 ± 28.84	120.3 ± 21.86
	Hardness	93.93 ± 8.879	86.70 ± 6.436

## Data Availability

The data presented in this study are available on request from the corresponding author.
